# Faster detection of asymptomatic COVID-19 cases among care home staff in England through the combination of SARS-CoV-2 testing technologies

**DOI:** 10.1038/s41598-024-57817-1

**Published:** 2024-03-29

**Authors:** Finola Ryan, Joanna Cole-Hamilton, Niharika Dandamudi, Matthias E. Futschik, Alexander Needham, Rida Saquib, Raghavendran Kulasegaran-Shylini, Edward Blandford, Michael Kidd, Éamonn O’Moore, Ian Hall, Malur Sudhanva, Paul Klapper, Andrew Dodgson, Adam Moore, Madeleine Duke, Sarah Tunkel, Chris Kenny, Tom Fowler

**Affiliations:** 1 Public Health and Clinical Oversight (PHCO), Clinical and Public Health Group, UK Health Security Agency, 10 South Colonade, Canary Wharf, London, E14 4PU UK; 2 King’s College Hospital NHS Foundation Trust, London, UK; 3 Division of Surgery & Interventional Science, University College London, London, UK; 4 School of Biomedical Sciences, Faculty of Health, University of Plymouth, Plymouth, UK; 5 UKHSA Regional Laboratory, Birmingham, UK; 6 Department of Mathematics, The University of Manchester, Manchester, UK; 7 Advanced Analytics, Analytics & Data Science, UK Health Security Agency, London, UK; 8 Clinical Virology, Division of Evolution, Infections and Genomics, University of Manchester, Manchester, UK; 9 Operational Policy, UK Health Security Agency, London, UK; 10 Queen Mary University of London William Harvey Research Institute, London, UK; 11 National Health Protection Office, HSE, Dublin, D01 A4A3 Ireland

**Keywords:** Viral infection, Infectious-disease diagnostics, Policy and public health in microbiology, SARS-CoV-2

## Abstract

To detect SARS-CoV-2 amongst asymptomatic care home staff in England, a dual-technology weekly testing regime was introduced on 23 December 2020. A lateral flow device (LFD) and quantitative reverse transcription polymerase chain reaction (qRT-PCR) test were taken on the same day (day 0) and a midweek LFD test was taken three to four days later. We evaluated the effectiveness of using dual-technology to detect SARS-CoV-2 between December 2020 to April 2021. Viral concentrations derived from qRT-PCR were used to determine the probable stage of infection and likely level of infectiousness. Day 0 PCR detected 1,493 cases of COVID-19, of which 53% were in the early stages of infection with little to no risk of transmission. Day 0 LFD detected 83% of cases that were highly likely to be infectious. On average, LFD results were received 46.3 h earlier than PCR, enabling removal of likely infectious staff from the workplace quicker than by weekly PCR alone. Demonstrating the rapidity of LFDs to detect highly infectious cases could be combined with the ability of PCR to detect cases in the very early stages of infection. In practice, asymptomatic care home staff were removed from the workplace earlier, breaking potential chains of transmission.

## Introduction

Care home residents tend to be more vulnerable to COVID-19 due to factors such as older age and multiple long-term health conditions as well as factors related to the settings themselves, such as shared living spaces and staff circulating between high-risk individuals. To minimise transmission to this group, the need for timely identification of care home staff infected by SARS-CoV-2, the virus responsible for COVID-19, was recognised early in the pandemic^1^. In England, testing of symptomatic staff began in mid-April 2020. It later became clear that asymptomatic transmission was possible^2^, and weekly testing by quantitative reverse-transcription polymerase chain reaction (qRT-PCR, referred to as PCR from here on) was introduced for asymptomatic adult social care (ASC) staff in England. When lateral flow device (LFD) antigen tests became available for use in the UK, the weekly PCR test was supplemented with twice weekly LFD testing. The testing regime consisted of a weekly PCR test on the first day of that week’s shift pattern (known as day 0) plus a twice weekly LFD with one LFD taken on day 0 and one taken about three to four days later. This approach was introduced based on recommendations and modelling from the Scientific Advisory Group for Emergencies Social Care Working Group (SAGE SCWG), which advised that such approach would provide a greater level of risk mitigation than using either technology alone. Furthermore, this particular sequence of tests would provide a better balance of increasing protection whilst balancing resource needs. Those who tested positive were restricted from the workplace and required to self-isolate and to co-operate with contact tracing. A similar form of the model used in the SAGE SCWG decision-making has been published^3^.

PCR tests have high sensitivity (85–98%^4^) with the ability to detect very low levels of RNA, whereas LFDs test for viral antigen, and thus detect a different kind of viral material. At the time when LFDs were being introduced into the UK testing programme there were concerns around their sensitivity in comparison to PCR tests. However, PCR tests may detect dead or unviable viral material and use of PCR for testing asymptomatic staff was limited by the required laboratory processing, associated costs and the turnaround time to receive a result. In contrast, LFDs can be used as self-tests for viral antigen that require no laboratory processing and can provide a quick result (within 30 min). These features might enable earlier removal of infectious asymptomatic staff from the workplace with the associated likely reduction in transmission^5^.

Studies have estimated the sensitivity of Innova LFDs (a brand of LFDs widely used in the UK during the pandemic) at 78.8%^6^, 61.3%^7^ or 64.3%^8^ when taking PCR tests as reference. It has been shown that symptom status affects the likelihood of an LFD to return a positive result^7–9^. Some studies have estimated sensitivity of Innova LFDs in asymptomatic test settings at a level of 50%^8^ or 40%^10^. Few have investigated the use of LFD as part of routine asymptomatic testing and those that have, estimated positive predictive values (PPV) but not sensitivity, as the testing regime did not include PCR testing to compare against^11,12^. Studies which have looked at care home testing regimes in England have considered adherence to the testing protocol and comparison between pilot and non-pilot care homes in terms of prevention of outbreaks^13^, or focused on the challenges of implementing such a scheme^14^ rather than effectiveness of the regime in detecting cases of SARS-CoV-2.

Here we present the results of a retrospective evaluation of the dual-technology testing regime and the benefits of including LFDs within asymptomatic testing for care home staff in England between 24 December 2020 and 28 April 2021.

## Methods

### Study design

**Figure 1:**
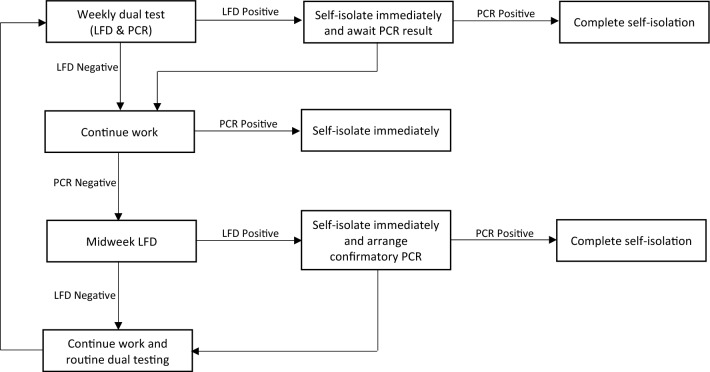
Description of the asymptomatic dual testing regime in adult social care. On the first day of the shift pattern the day 0 LFD and PCR tests were taken. Three to four days later a midweek LFD test was taken. A day 0 LFD positive test result led to self-isolation until the PCR result was returned. A midweek LFD positive led to self-isolation and the individual was advised to take a confirmatory PCR. The result of the PCR determined if the staff member completed the full 10 days of self-isolation or returned to work.

ASC staff in England took part in routine asymptomatic testing each week as shown in Fig. 1. Dual testing (LFD and PCR) was undertaken on the first day of that week’s shift pattern, known as day 0. The midweek LFD was taken three to four days after the day 0 tests. Each care home organised testing in accordance with UK Government guidance and as determined by their individual capacity. Day 0 LFD and PCR were taken on the same day though not necessarily at the same time. Anecdotally, in several settings, LFD tests were conducted at home and PCR at the place of work from where the swabs were then sent to the lab. Each individual staff member self-swabbed for both the LFD and PCR. For the LFD, the individual staff member carried out and interpreted the test themselves. The result of the LFD test may have been known prior to swabbing for PCR.

The LFD used in ASC was the *Innova COVID-19 Self-test (Rapid Antigen Test),* used in accordance with the instructions for use^15^. Swabbing was performed on the nose and throat and the wait time to read the test was 30 min. LFD results were dichotomous (positive or negative) therefore the categories were pre-specified. Void test results were not reported, and the test was repeated to obtain a non-void result. On receiving a positive result, the staff member was instructed not to attend work or immediately leave the workplace and remain at home in self-isolation for 10 days, as per UK Government requirements at the time. If the midweek LFD was positive, they were asked to take a confirmatory PCR test. If the day 0 or confirmatory PCR was negative, the staff member could leave self-isolation, return to work and continue routine testing.

PCR swabbing was performed on the nose (bilateral mid-turbinate) and throat. The PCR results were classified as positive, negative, or void by the laboratory. If the PCR test indicated a positive result, the person was asked to self-isolate for 10 days. If negative, the person continued to work. If void, the PCR test was repeated. Staff or care home administrators entered the LFD results into the national digital reporting service. To alleviate administrative pressure, for the first four weeks of the policy, care homes could opt out of registering all LFD results. PCR test results were uploaded to the National Pathology Exchange (NPEx) database by the laboratories.

The testing undertaken in care homes was part of the UK national pandemic response and the testing regimes were determined by national policy. In this study we accessed data from the testing programme to evaluate its impact as part of the Public Health response. As such, informed consent was not required from individuals whose test results were used. This was confirmed by use of the Health Research Authority (HRA) research tool and after review by the UK Heath Security Agency (UKHSA) Research Support and Governance Office**.** All relevant guidelines and regulations were followed for testing of subjects, data extraction and data analysis.

### Data extraction

PCR and LFD test results for all ASC staff were extracted from NPEx and then filtered to extract only results for care home staff from 24 December 2020 to 28 April 2021, the start of the testing policy to the date of first analysis of data (though results were monitored on an ongoing basis). This roughly corresponds with the period during which the Alpha variant was dominant^16^. LFD and PCR test results were matched to unique individuals through a strict matching method using personal identifiers such as first name, last name and date of birth. Consequently, if a staff member entered their name differently on separate occasions they would not have been matched. Where matching criteria was not met, data was excluded. Data was then pseudo-anonymised, and this pseudo-anonymised dataset was accessed for the current analysis. Excluded from the dataset were: all those who reported symptoms at the time of testing; any day 0 PCR test processed in a laboratory for which the conversion formula from cycle quantification (Cq) to viral concentration was not known; participants with reported ages outside that of normal working age (18–65).

### Statistical analysis

For the purpose of this paper, viral concentration is defined as the number of severe-acute-respiratory-syndrome-related coronavirus-2 (SARS-CoV-2) ribonucleic acid (RNA) viral copies present per mL of viral transport medium. The number of cases detected by PCR was determined, and Cq values of positive PCR samples were converted to viral concentation in copies/mL using the laboratory and gene specific conversion formulae provided by the laboratories (as published in Ref.^8^). This is a proxy for the amount of virus present in a person’s nasal or oral cavity rather than a direct measure. It depends both on the quality of the swabbing technique and the efficiency of the release of the virus from the swab into the transport medium. Other reports and papers may refer to this as viral load or burden. While there is no specific viral concentration below which someone will definitely not be infectious, and above which they definitely will, for the purposes of this analysis, PCR positive results were grouped according to how likely the individual was considered to be infectious^17,18^. Below 10,000 copies/mL was considered to be of negligible or no infection risk; 10,000–1million copies/mL was considered to be of little infection risk; and above 1million copies/mL was considered highly likely to be infectious.

Assuming that PCR tests present a gold standard with perfect sensitivity, the Day 0 PCR results were used as the reference against which day 0 LFD performance was measured overall and over time (each month). The number of true positive (TP) was defined as the number of positive LFDs with matched positive PCR tests; the number of false positive (FP) as positive LFDs with matched negative PCR tests; the number of true negative (TN) as number of LFD negative with PCR negative; and the number of false negative (FN) as the number of LFD negative matched with PCR positive tests. Sensitivity of LFDs on day 0 was calculated for the three viral concentration categories mentioned above using the formula: Sensitivity = TP/(TP + FN). Specificity was calculated as specificity = TN/(TN + FP). PPV was defined as PPV = TP/(TP + FP). Along with PPV, modelled PPV was also calculated to account for positivity rate which is known to affect PPV; modelled PPV was defined as, modelled PPV = (positivity * sensitivity) / ((positivity * sensitivity) + (1-specificity) * (1-positivity)), where positivity = (TP + FN)/(TP + FN + FP + TN) [i.e. PCR + /(PCR −  + PCR +)]. Negative Predictive Value (NPV) was calculated as NPV = TN/(TN + FN). The Clopper-Pearson exact method was used to derive 95% confidence intervals (CI). To assess the benefit in including an LFD on the same day as PCR in the regime, the time between receiving the LFD result and PCR result was calculated for day 0 results.

Midweek LFD positives were matched, where possible, to a PCR taken by the same individual within six days of the LFD result (it was presumed that any later than this was more likely to be the next day 0 PCR). This was assumed to be a confirmatory PCR and was used to assess performance of the standalone midweek LFD. Midweek LFD results were not included in the above calculations of sensitivity or other statistical measures, since the confirmatory PCR was not taken on the same day.

### Ethics approval and consent to participate

Within the context of the pandemic public health response and roll out of testing interventions, after review using the HRA tool and after further discussions with HRA it was determined that this evaluation would not require HRA research ethics approval. All study participants received routine care through receipt of an individual diagnostic swab test and result. This protocol was further reviewed by the Research Support and Governance Office and classified as work that was undertaken as part of PHE’s (Public Health England, and its successor organisation UK Health Security Agency) responsibility to respond to the COVID-19 pandemic. PHE had legal permission, provided by Regulation 3 of The Health Service (Control of Patient Information) Regulations 2002, to collect confidential patient information (http://www.legislation.gov.uk/uksi/2002/1438/regulation/3/made) under Sections 3(i) (a) to (c), 3(i)(d) (i) and (ii) and 3(3) as part of its outbreak response activities. As such this work fell outside the remit for ethical review and as no regulatory issues were identified the protocol was approved.

## Results

After exclusions, there were 1,738,893 tests identified that were taken by asymptomatic care home staff as part of the testing regime in the period examined; 189,364 care home staff took 644,480 dual-tests (LFD and PCR) plus 449,933 mid-week LFDs. Of the dual tests, over half (56%) were taken on a Monday or Tuesday, with only 4% taken at the weekend. Just under half (48%) of midweek LFDs were taken on a Thursday or Friday. It was most common to take the midweek LFD three days (39.8%) or four days after (28.5%) after the day 0. The age range was 18–65 with the median age of 45, first quartile of 33, third quartile of 55 and an interquartile range of 22. Of the study cohort, 83% were female.

There were 1,493 PCR positive results giving a positivity rate of 0.2% in the cohort studied. The total number of PCR results and positive results by viral concentration category for each month and the whole study period are shown in Table 1. Of 1493 cases of SARS-CoV-2 detected by PCR, 341 (22.8%) had a viral concentration in the highest category (> 1M copies/mL), while 790 (52.9%) had a viral concentration in the lowest category (< 10,000 copies/mL). Thus, 1,152 of the 1,493 positive cases detected by PCR (77.2%) were not considered highly infectious at the time of testing.

**Table 1:** PCR tests taken, positive results by viral concentration category, and positivity rate each month.

	PCR + > 1M copies/mL	PCR + 10K – 1M copies/mL	PCR + < 10K copies/mL	PCR-positive Total	PCR-negative Total	Total dual tests	Test positivity %
Dec-20	3	10	7	20	1334	1354	1.48
Jan-21	221	162	323	706	87,577	88,283	0.80
Feb-21	76	108	221	405	149,042	149,447	0.27
Mar-21	33	71	145	249	217,876	218,125	0.11
Apr-21	8	11	94	113	187,158	187,271	0.06
Total tests	341	362	790	1493	642,987	644,480	0.23

Day 0 results referenced against day 0 PCR; midweek LFD results referenced against the next PCR (within 6 days).

In total, 755 day 0 LFD positives and 387 midweek LFD positives were recorded. With respect to PCR as reference, the day 0 LFD results comprised 440 TPs, 315 FP, 1,053 FNs and 642,672 TNs. The LFD sensitivity at each viral concentration category is shown in Table 2. For a viral concentration over 1M copies/mL, a sensitivity of 83.0% was reached. Of the PCR-positive cases detected by LFD, 64.3% were in the highly infectious viral concentration category. Of the 58 PCR positive/LFD negative cases in the highly infectious category at day 0, 13 had a viral concentration > 10M copies/mL. The median viral concentration in each viral concentration category was skewed towards the lower end of the category, particularly in the two least infectious categories (Table 3).

**Table 2:** PCR positive LFD results by viral concentration.

Viral concentration (copies/mL)/infectiousness	Day 0 LFD TP	Day 0 LFD FN	Day 0 sensitivity % (95% CI)	Midweek LFD positive/Next PCR positive	Midweek LFD negative/Next PCR positive
< 10,000 (negligible or no infection risk)	18	772	2.3 (1.4–3.6)	2	49
10,000–1 M (little infection risk)	139	223	38.4 (33.4–43.6)	14	6
> 1 M (highly likely to be infectious)	283	58	83.0 (78.6–86.8)	47	5
Total tests:	440	1053	29.5 (27.2–31.9)	63	60

**Table 3:** Median viral concentration in each viral concentration category by month.

Category	Month	Median viral concentration (copies/mL)
Highly likely to be infectious (> 1 M copies/mL)	Dec-20	8.7 M
	Jan-21	7.7 M
	Feb-21	4.2 M
	Mar-21	5.6 M
	Apr-21	9.5 M
Little infection risk (10 K-1 M copies/mL)	Dec-20	122 K
	Jan-21	170 K
	Feb-21	104 K
	Mar-21	87 K
	Apr-21	203 K
Negligible or no infection risk (< 10 K copies/mL)	Dec-20	9
	Jan-21	73
	Feb-21	74
	Mar-21	100
	Apr-21	85

**Figure 2:**
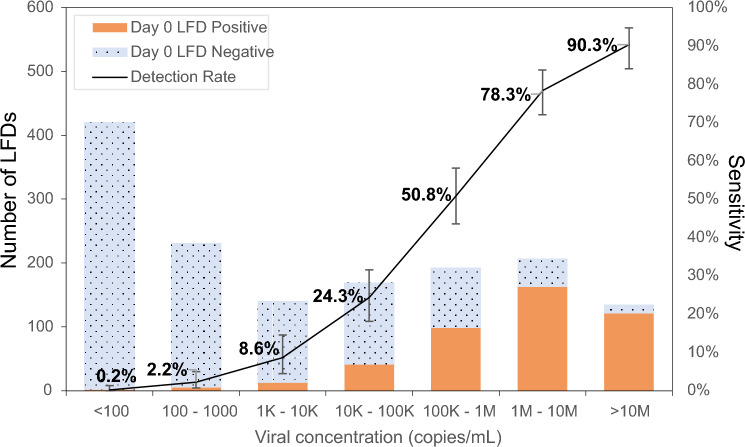
Day 0 LFD performance for seven narrow categories of viral concentration. Day 0 PCR was used as comparison to determine TP and FN.

When narrower viral concentration categories (log_10_ intervals) were used, the effect of viral on LFD sensitivity was clearly seen to increase from 0.2% (0–1.3%) at < 100 copies/mL to 90.3% (84.0–94.7%) at > 10M copies/mL (Fig. 2). Figure 2 also shows that the viral concentration category most commonly detected by PCR was < 100 copies/mL for which LFDs would not be expected to detect antigen; the least commonly detected viral concentration was > 10M copies/mL. As viral concentration increased the proportion of PCR positive cases detected by LFD increased.

**Figure 3:**
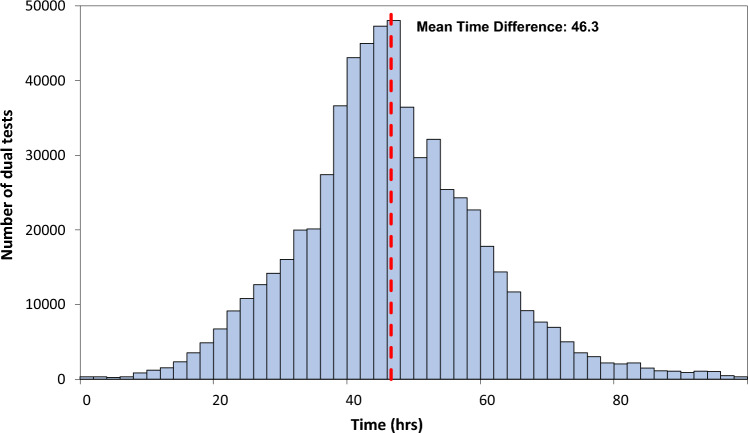
Distribution of time between receiving the day 0 LFD and day 0 PCR results. Dashed red indicates mean time difference.

The time between receiving the day 0 LFD result and PCR result is approximately a normal distribution with a mean of 46.3 h (SD: 13.8) (Fig. 3). The median time dropped from more than 60 h at the beginning of January to around 45 h in February and March and fell again to around 35 h in April.

LFD specificity was high with 99.95% (99.95–99.96%) over the observed period. PPV fell steadily each month from 84.6% (54.6–98.1%) in December, to 14.3% (7.6–23.6%) in April (Table 4). The positivity rate also fell over the same time period (Table 1) which corresponds with a reduction in the prevalence among the general population^19^. When PPV was modelled to the December positivity rate, the modelled PPV each month (Table 4) was similar. NPV over the observed period was 99.84% (99.83–99.85%) (Table 4).

**Table 4:** LFD performance results by month and overall performance.

Month	Sensitivity % (95% CI)	Specificity % (95% CI)	PPV % (95% CI)	NPV % (95% CI)	Adjusted sensitivity % (95% CI)	Modelled PPV using December positivity of 1.48% (95% CI)	Test positivity rate %
December 2020	55.0 (31.5–76.9)	99.85 (99.46–99.98)	84.6 (54.6–98.1)	99.33 (98.73–99.69)	47.8 (23.1–68.5)	84.6 (46.7–98.5)	1.48
January 2021	38.7 (35.1–42.4)	99.91 (99.89–99.93)	78.0 (73.3–82.2)	99.51 (99.46–99.55)	38.7 (35.2–42.5)	86.8 (82.7–90.2)	0.80
February 2021	24.7 (20.6–29.2)	99.95 (99.94–99.96)	56.8 (49.2–64.3)	99.80 (99.77–99.82)	34.3 (29.8–39.3)	87.9 (82.9–91.6)	0.27
March 2021	17.7 (13.1–23.0)	99.96 (99.95–99.97)	33.3 (25.4–42.1)	99.91 (99.89–99.92)	28.9 (23.4–35.0)	86.8 (79.8–91.4)	0.11
April 2021	10.6 (5.6–17.8)	99.96 (99.95–99.97)	14.3 (7.6–23.6)	99.95 (99.93–99.96)	34.8 (24.2–42.2)	80.5 (63.5–89.9)	0.06
Overall	29.5(27.2–31.9)	99.951 (99.945–99.956)	58.3(54.7–61.8)	99.84(99.83–99.85)	32.6(30.3–35.1)	90.0 (88.1–91.6)	0.23

We retrieved 387 positive midweek LFD results; 253 had a matched PCR within 6 days (159TP/94FP). Of these, 63 TP had a recorded Cq value which could be converted to viral concentration and are shown in Table 2; 47 of these TP were likely to be highly infectious (Table 2). 60 midweek LFDs returned a negative result, and the next PCR returned a positive; 49 (82%) of these PCRs had a viral concentration of below 10,000 copies/mL, 41 were below 1,000 copies/mL, and 29 were even below 100 copies/mL, indicating very new infections that may not have been present at the time of the midweek LFD. Of the 5 with viral concentration > 1M copies/mL, two took a PCR 2 days after the LFD, and three took it 4 days after. 75% of the midweek LFD positives confirmed as positive by PCR had a viral concentration > 1M copies/mL, 22% had a viral concentration of 10,000-1M copies/mL, and 3% had viral concentration < 10,000 copies/mL, compared to 64%, 32% and 4% respectively for the day 0 LFD positives confirmed as positive by day 0 PCRs.

## Discussion

A total of 1,493 cases of SARS-CoV-2 were detected by PCR in the dual-technology testing regime of which 790 (53%) were in the lowest infection risk viral concentration category of < 10,000 copies/mL. Low viral concentration is observed at the beginning and end of the infection cycle. Given that staff were participating in regular testing, it is likely most of these cases were in the early stages of infection. The growth phase of the infection cycle has been estimated at 3.6 days for viral RNA shedding and 1.6 days for infectious viral shedding^20^. It is possible that returning the results of PCR positive cases with viral concentration below 10,000 copies/mL within the average 46 h of taking the test may have been soon enough to remove these care workers from the workplace prior to them having the potential to transmit, though the dynamics of infectious viral shedding appear to be diverse^20^. It is also possible that these cases would have been detected by the next LFD in the testing regime three to four days later at the end of the growth phase of the infection cycle when the viral concentration was high.

Of those cases detected by PCR that were highly likely to be infectious, LFDs detected 83%, removing the staff member from the workplace, on average, 45 h before the PCR result was returned.

Midweek LFDs detected a further 159 cases of COVID-19 that were later confirmed by a PCR test. Of the 63 confirmed by PCR with recorded Cq value, 47 (74.6%) were highly likely to be infectious suggesting that a midweek LFD was an appropriate approach to pick up those who rapidly developed infection and posed the highest risk of transmission. Without the midweek LFD these individuals would not have been identified until the next day 0 tests three to four days later. Given the similar viral concentration distribution of midweek and day 0 LFD positives, it might be assumed that the midweek LFDs detect a similar proportion of cases at each viral concentration category as the day 0 LFDs and that there is a similar time between midweek LFD and receiving the confirmatory PCR result (i.e. 46.3 h).

Performance of LFDs in our study broadly aligns with others who have found that sensitivity increased as viral concentration increased^6,7,9^, though sensitivity in each viral concentration category was slightly lower than that found by other studies of asymptomatic testing. The sensitivity for Innova LFD in the dual-testing regime was 2.3% at viral concentration < 10,000 copies/mL; 38.4% at viral concentration of 10,000-1M copies/mL; and 83% at viral concentration > 1M copies/mL. Mass asymptomatic testing using Innova LFDs in Liverpool observed sensitivities of 90.9% (58.7–99.8%) for a viral concentration > 1M copies/mL, 69.4% (51.9–83.7%) for a viral concentration > 10,000 copies/mL (includes > 1M category), and 9.7% (1.9–23.7%) for a viral concentration < 10,000 copies/mL (10). As may be expected in a routine testing regime, viral concentration distribution was skewed towards the lower end of each category (Table 3) which may explain some of this reduction in sensitivity, especially in the lower category where the median viral concentration was ≤ 100 copies/mL.

It is also possible that the testing regime itself contributed to a lower monthly sensitivity than LFD sensitivities previously derived in cross-sectional studies. The day 0 sensitivity calculation did not include those staff members who tested midweek LFD positive since the staff member would have stopped taking part in the dual-testing regime due to self-isolation. This thereby lowered the overall sensitivity of LFDs in this testing regime in comparison to a normal diagnostic accuracy study where a cross-section of the population would be tested and there would have been no prior testing to remove anyone from taking part.

Comparing concurrent (or approximately concurrent) results is likely to bias the sensitivity of LFD compared to PCR which will detect infection sooner though it is possible that with regular LFD testing on days 1, 2, and 3 for example would still have detected the case quicker than PCR due to the delay in receiving the result (see Fig. 3 in Ref.^21^).

The performance of LFDs has been shown to correlate closely with the presence of viral culture/plaque-forming units and to therefore give an indication of infectiousness at the time of testing^20,22,23^. LFDs have shown their lowest sensitivity in the viral growth phase of the infection cycle and highest sensitivity in the decline phase^20,22^. This difference in LFD sensitivity at different stages of the infection cycle appears to be occurring in the dual-testing regime given the low sensitivity observed in the early stages of the infection cycle when viral concentration was low. This supports the use of dual technology in asymptomatic testing regimes which can detect both those in the early stages of infection (by PCR), and also those who are highly likely to be infectious at the time of testing and to rapidly return a result (by LFD) which can be acted on almost immediately.

This study had some limitations such as the strict matching method for PCR and LFD results mentioned in the methods section which among other criteria would have excluded staff members that entered their personal details differently on separate occasions. It is likely that the number of tests identified here as being taken as part of the ASC testing regime is an underestimate not only due to the matching method, but also because care homes had to register the LFD results themselves and may not always have done so due to the administrative burden on staff (PCRs results were registered by the lab and will always have been reported). Furthermore, for the purposes of this study an assumption was made that PCR tests are accurate 100% of the time; however, they can also provide inaccurate results. Finally, PCR and LFD swabs were not necessarily taken in immediate succession which has the potential to lead to discordant results that are both, in fact, correct.

The strengths of the study include the large sample size and the unique occupational population examined. It is likely that the results of this study are transferable to other healthcare workers and similar settings. Furthermore, the evaluation provides an estimate of LFD sensitivity as used in a real-world routine asymptomatic testing regime.

## Conclusion

This evaluation has shown that combining two different types of testing technology is an effective way of testing asymptomatic staff working with vulnerable individuals. The dual testing regime with PCR and LFD can enable quick identification of highly infectious cases as well as those staff who are in the early stages of infection. Near-person testing with lateral flow technology is simple and inexpensive and delivers a result within minutes rather than days. This has important implications for future pandemic testing policy; although one test may be more sensitive, supplementing that test with another that assesses different viral material can prove beneficial in detecting more cases or cases more quickly. Further research is required to determine the effectiveness of other asymptomatic testing regimes and the most effective combination of tests.

## Data Availability

The datasets used and analysed during the current study are available from the corresponding author on reasonable request.
